# Supplementary material for: Role of FAM134 paralogues in endoplasmic reticulum remodeling, ER‐phagy, and Collagen quality control
Source: EMBO Rep. 2021 Aug 2;22(9):e52289. doi: 10.15252/embr.202052289 (PMC8447607; doi:10.15252/embr.202052289)
Supplement: Supplementary file 2 — Expanded View Figures PDF [file EMBR-22-e52289-s014.pdf]

Expanded View Figures

Figure EV1. Biophysical properties and tissue expression levels of FAM134 proteins.

A Alignment of the amino acid sequence of human FAM134 proteins. Blue, red, and yellow box highlights the reticulon homology domain (RHD), the LIR domains, and predicted α-helixes adjacent to the LIR domain, respectively ("*" no mismatches among sequences; more or less conserved amino acidic groups are highlighted with "." and ":", respectively).

B Immunofluorescence images of U2OS cells overexpressing FLAG-HA-FAM134 proteins after 24 h doxycycline induction and stained for FAM134 (HA) or endogenous REEP5. Cells grown in basal or starvation (2 h EBSS treatment) conditions and 200 nM Bafilomycin A1 (Baf.A1) was added 2 h prior fixation. Scale bar: 20 μm.

C Representative Western blot analysis of Fam134 proteins in different murine tissues. Wild-type and respective knockout MEFs served as internal control for antibody staining. An independent Ponceau staining is shown as loading control.

D Matrix representing the pairwise sequence identities (upper triangle; blue; parentheses show % similarities) and structural similarities (RMSD; lower triangle; gray) of the modeled RHD domains of the three human paralogs.

E Helical wheel representation of the linker regions AH_L and AH_C from RHDs of human FAM134 describing the relative orientation of the helices on the bilayer-water interface. The mean hydrophobicity, and hydrophobic moments are depicted on either side of the helical wheel along with net charge at its center (amino acids with a positive charge [R, K, H] are reported in blue; amino acids with a negative charge [E, D] are reported in red; amino acids with a hydrophobic side chain [M, L, I, Y, F, W, V, C] are depicted in yellow; alanine [A] is depicted in gray).

F Root mean square fluctuations (RMSF) of the RHD residues around their mean position after fitting backbone beads indicate that the cytosolic loops and the nonhelical RHD elements contribute to the differences in wedge-shape (gray: TM segments; yellow: AH segments).

G Distribution of hydrophobic moments and the hydrophobicity of FAM134 paralogues (orange contours) and canonical RTN4 (blue contours). FAM134A, FAM134B, and FAM134C are representative and shown as colored points.

H Comparison of the curvature induction process in bicelle systems containing FAM134 proteins. Data from curvature time-series of each system (n = 20 independent computational runs) were first smoothed over an 11-ns window and binned along the curvature to compute the average (black line) times + s.e.m. (shaded region).

I Violin plots showing the distribution of waiting times from n = 20 independent computational runs for bilayer-to-vesicle transitions for bicelles containing FAM134A-RHD (red), FAM134B-RHD (green) and FAM134C-RHD (blue). Each boxplot graph reports the median value with the first and third quartile of the distribution.

Source data are available online for this figure.

© 2021 The Authors

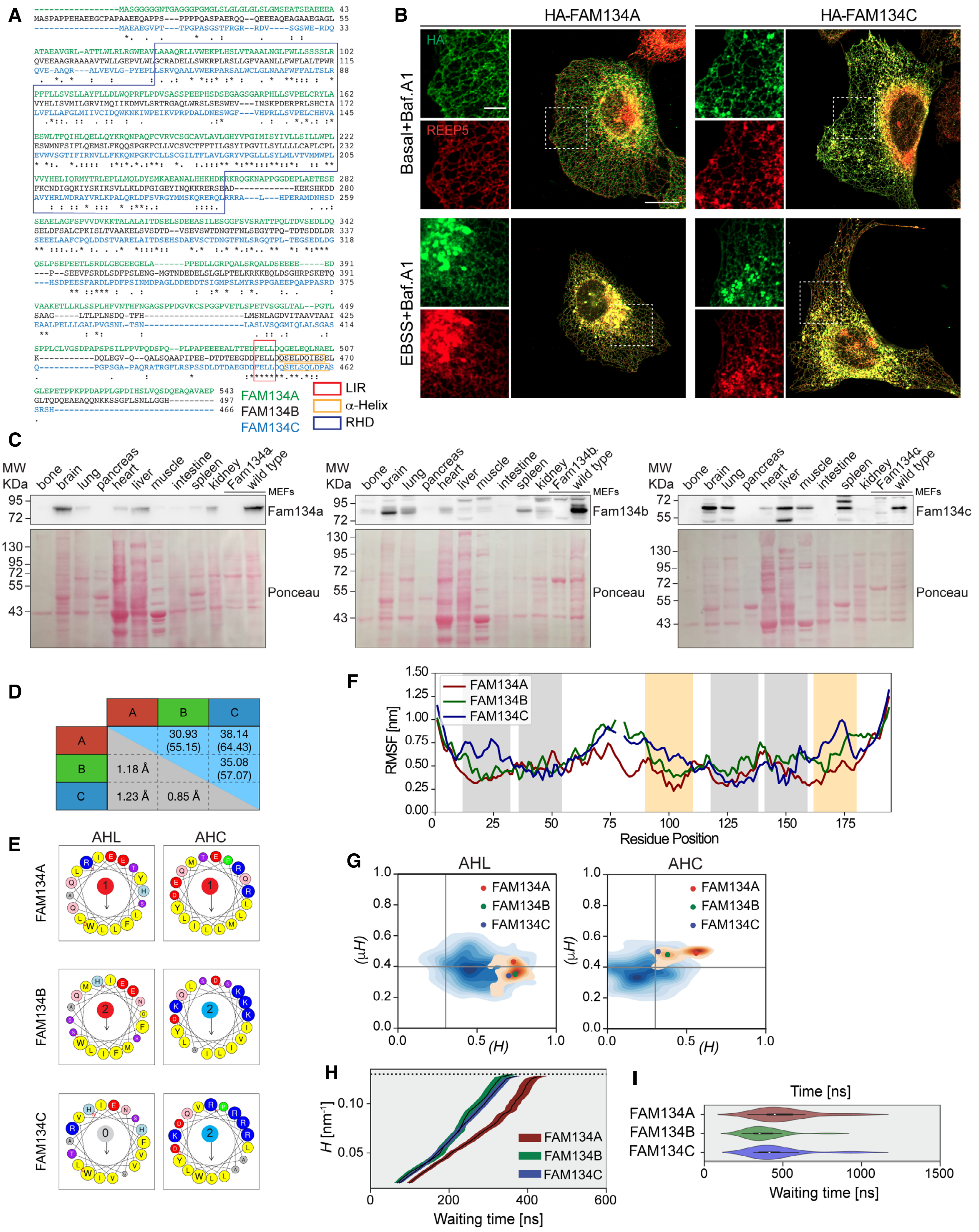

Figure EV1.

 © 2021 The Authors

Figure EV2. FAM134 proteins induce ER fragmentation at different levels.

A Lysates of wild-type MEFs were subjected to pulldown experiments (representative data of $n = 3$ independent cell preparation; WB detection against endogenous Fam134 using purified GST-LC3A/B and GST-GABARAPL1/L2 as baits. A representative Ponceau staining is shown.

B Representative immunofluorescence images of U2OS expressing wild-type or ΔLIR mutant FLAG-HA-FAM134 proteins after 24 h of doxycycline induction under different conditions (basal = DMSO, Baf.A1 = 2 h of 200 nM Bafilomycin A1, EBSS = 2 h of starvation in EBSS). Scale bar: 10 μm. Inset scale bar: 5 μm; staining against FAM134 (HA; green).

C Statistic analysis of dot-like structures and ER branching of images presented in (B). Automatic quantification of HA-positive dot-like structures and automated quantification of ER branching as described in Valente *et al* (2017). Each data point represents the average of dots per cell of one view (representative image see Fig EV2B). n.s. = not significant. FBS (Fetal Bovine Serum): indicates DMEM with 10% FBS.

D Immuno-gold labeling of HA in U2OS FLAG-HA-FAM134-overexpressing cells after 2 h EBSS starvation plus 200 nM Bafilomycin A1. AV: autophagic vesicles; ER: endoplasmic reticulum. Scale bar: 500 nm.

E Representative immunofluorescence images of U2OS cells expressing wild-type or ΔLIR mutant FLAG-HA-FAM134 proteins after 24 h of doxycycline induction under different conditions (basal = DMSO, Baf.A1 = 2 h of 200 nM Bafilomycin A1, EBSS = 2 h of starvation in EBSS). Scale bar: 10 μm; staining against FAM134 (HA; green) and endogenous LAMP1 (red).

F Representative Western blot of FAM134 protein levels (HA) in U2OS cells expressing FLAG-HA-FAM134 after 24 h of doxycycline induction and EBSS starvation for the indicated time (h = hours).

G Densitometric analysis reporting the fold decrease of FLAG-HA-FAM134 expression level upon EBSS starvation for the indicated time (hours). Actin has been used as reference for ratio calculation. Data are represented as mean ± s.e.m. of three independent biological experiments and the statistical significance is calculated by unpaired t-test and defined as $^{*}P < 0.05$, $^{**}P < 0.01$, $^{***}P < 0.001$.

Source data are available online for this figure.

© 2021 The Authors

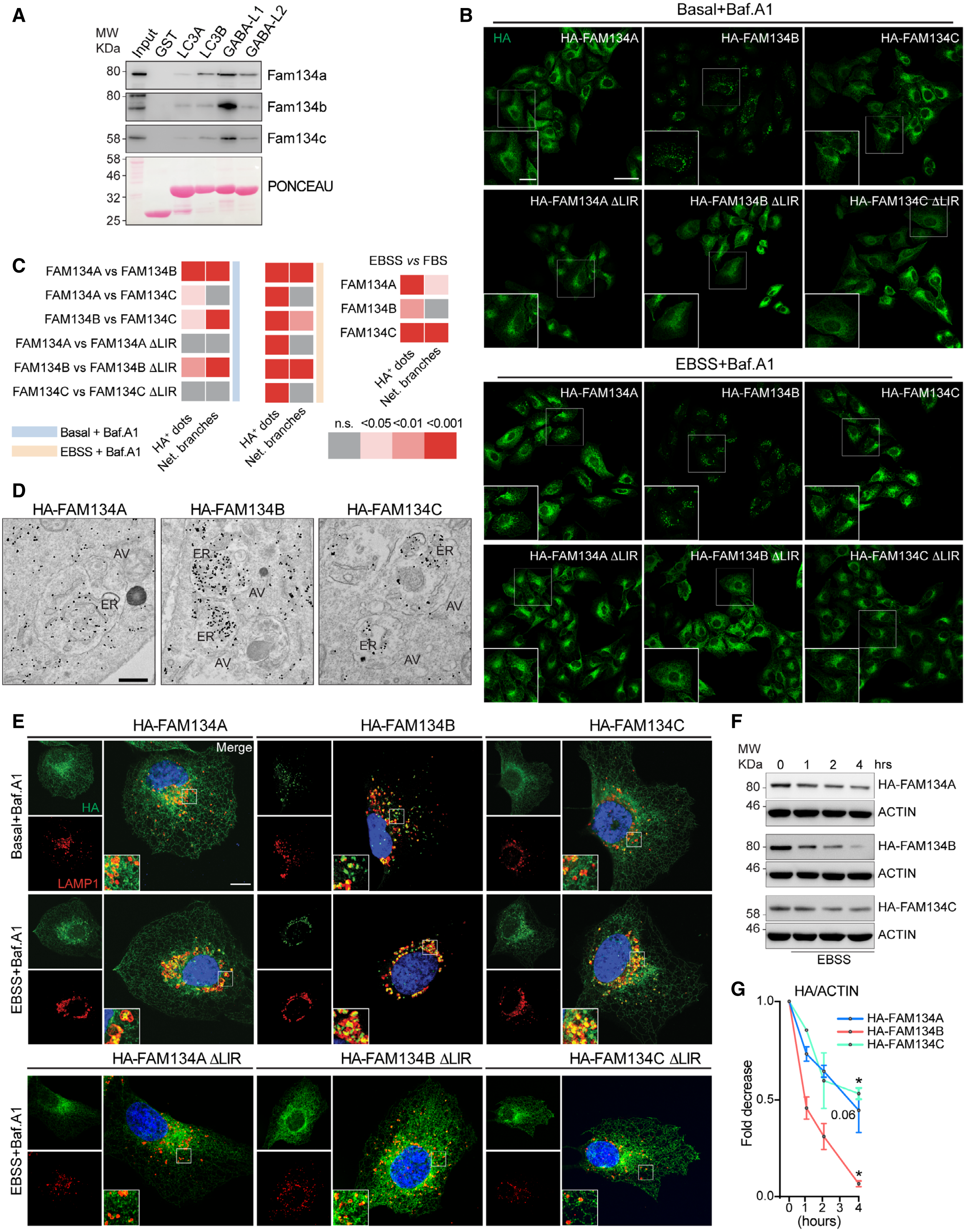

Figure EV2.

 © 2021 The Authors

Figure EV3. FAM134-positive ER fragments are delivered to lysosome via ER-phagy.

Immunofluorescence images of U2OS cells expressing FLAG-HA-FAM134 proteins after 24 h doxycycline induction were stained for FAM134 (HA), CANX, and LAMP1. Cells were grown in basal conditions or starved for 2 h with EBSS. Bafilomycin A1 was added 2 h prior fixation at the concentration of 200 nM. Scale bar: 10 µm. Inset scale bar: 5 µm.

© 2021 The Authors

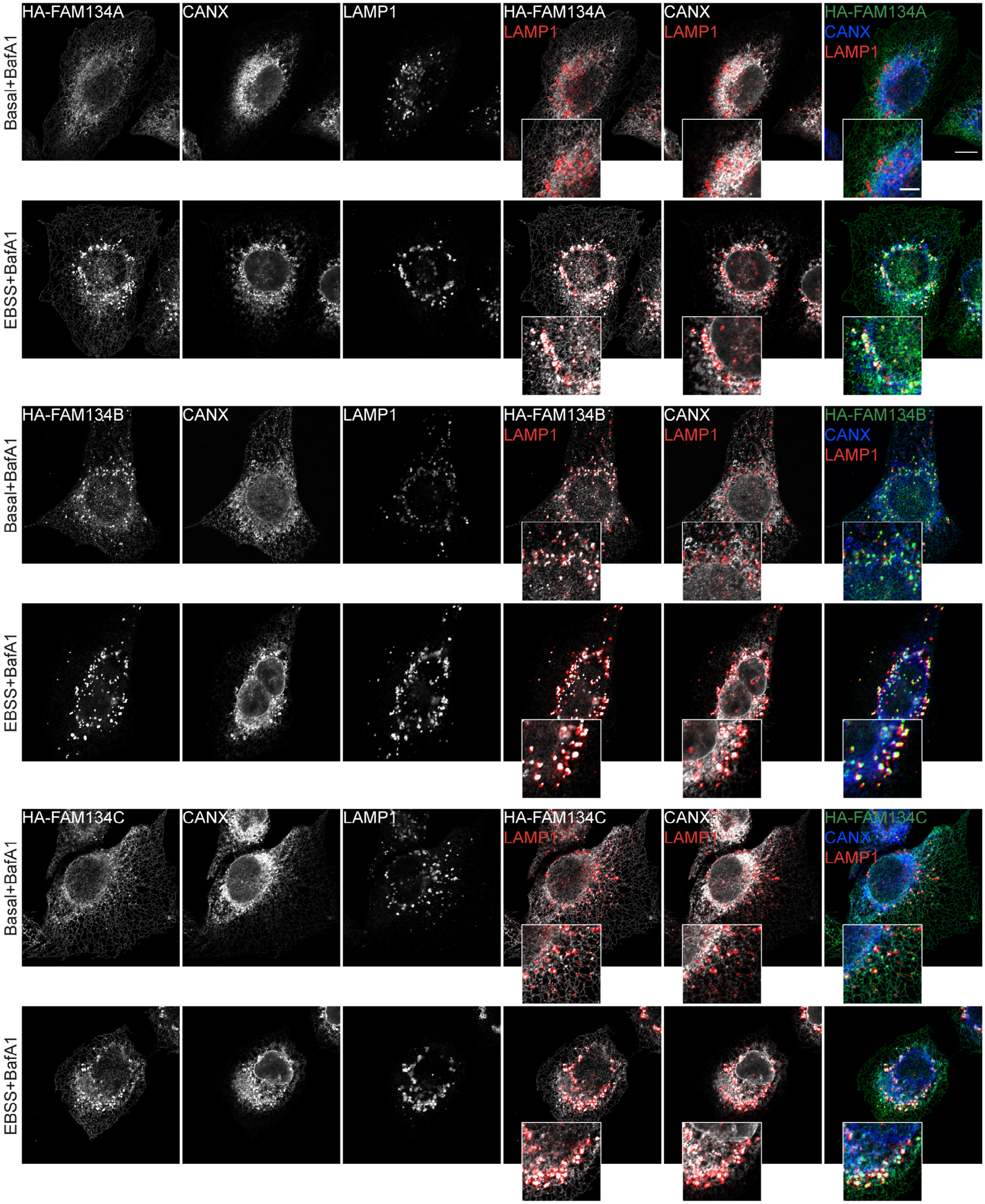

Figure EV3.

© 2021 The Authors

Figure EV4. Full proteome analysis in Fam134 knockout MEFs.

A Graphical representation of the analyzed proteomes. Numbers indicate the total identified proteins.

B Hierarchical clustering of label-free quantitation (LFQ) intensities of the identified proteins in the total proteomes of *Fam134s* single knockout MEFs compared to wild-type.

C Volcano plot of the global proteome analysis of *Fam134* single knockout MEFs compared to wild-type cells. Proteins with Log_2 Difference ≥ 1 and $-\text{Log}_{10}$ *P*-value > 1.3 (adjusted *P*-value 0.05) were considered significantly enriched. Group comparison has been performed by *t*-test statistics and adjusted for the false discovery rate (0.05 *P*-value adj).

D Venn Diagrams of up- and down-regulated proteins in *Fam134* single knockout MEFs compared to wild-type cells.

E Profile plots indicating protein levels (LFQ intensities) within clusters of identified protein presented in Fig 5D. GO cellular component (GOCC) terms are indicated together with the total number of identified proteins for each cluster.

F Insets showing the stress fibers network from Fig 5E.

G Heatmap of label-free quantitation (LFQ) intensities of significantly changed proteins within cluster8 (Fig 5D), with the GO terms: Collagen and endoplasmic reticulum.

H Representative Western blot for Collagen I, Vinculin and Actin from wild-type and knockout MEF cell lysates (IN) and culture media (OUT). Min indicates time points of samples collection.

Source data are available online for this figure.

© 2021 The Authors

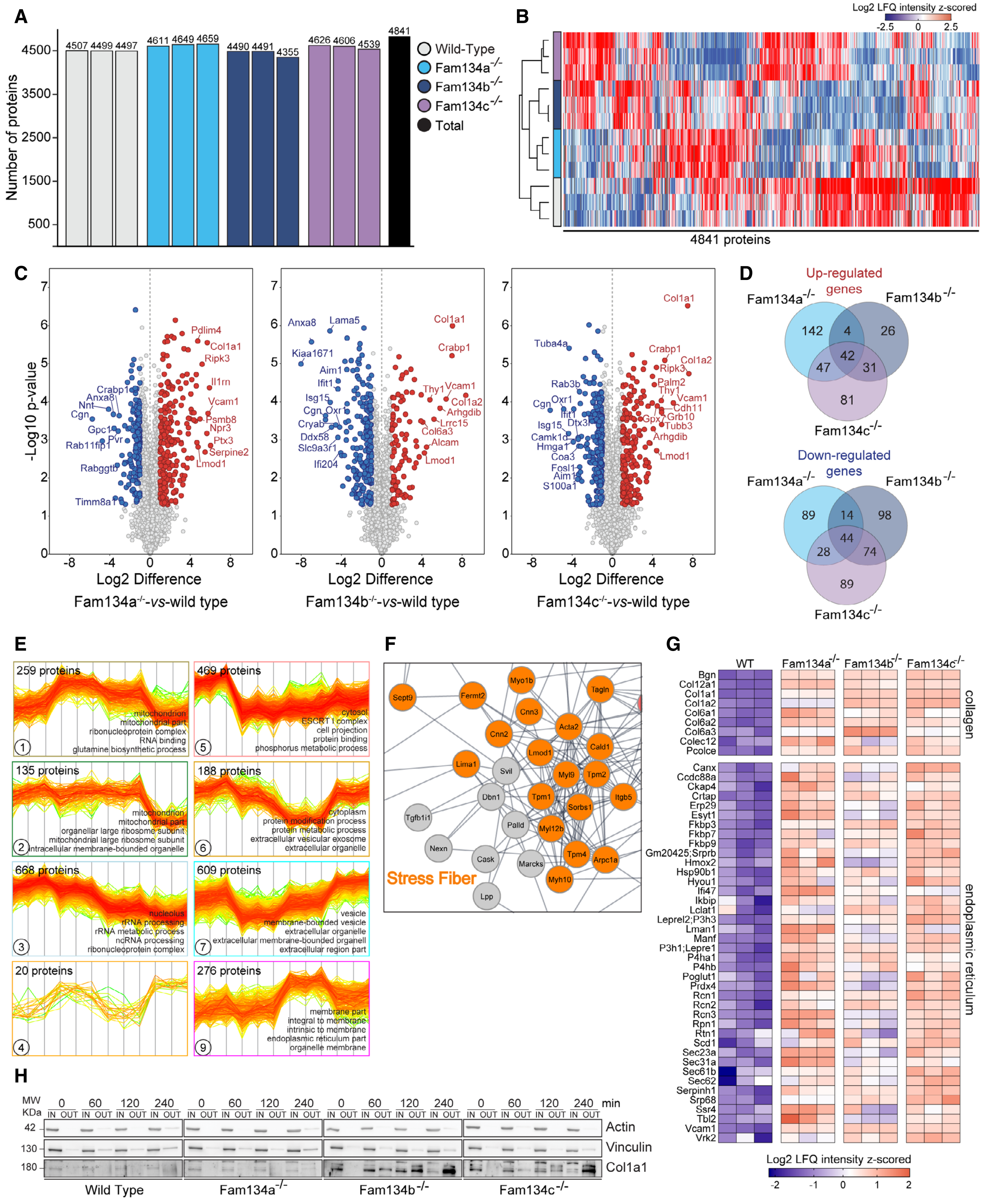

Figure EV4.

 © 2021 The Authors

Figure EV5. Fam134s knockout MEFs accumulate misfolded pro-Collagen I.

A Immunofluorescence images stained for endogenous Collagen I (red) and Lamp1 (green) in wild-type, *Fam134* knockout MEFs, and *Fam134* knockout MEFs reconstituted with the respective wild-type or ΔLIR mutant Fam134 protein. Scale bar: 10 μm. Inset scale bar: 5 μm.

B, C Co-Immunoprecipitation (Co-IP) experiment of FAM134 (HA; bait) and CANX in lysates of U2OS cells overexpressing FLAG-HA-FAM134 wild-type (B) or ΔLIR mutant (C) proteins.

D Co-IP experiment of endogenous CANX (bait) and FAM134 in lysates of U2OS cells overexpressing FLAG-HA-FAM134 wild-type proteins.

E Western blot of total cell lysates derived from *Fam134b* knockout MEFs and *Fam134b* knockout MEFs overexpressing the wild-type or ΔLIR mutant of Fam134a.

F Co-IP experiment of FAM134 (HA; bait) and endogenous LC3B in lysates of U2OS cells overexpressing indicated FLAG-HA-FAM134 proteins and grown under basal or starvation (2 h EBSS) conditions. 200 nM Bafilomycin A1 was added 2 h prior cell lysis.

G Co-IP experiment of FAM134 (HA; bait) and endogenous LC3B (upper panel) or GABARAPs (lower panel) in lysates of U2OS cells overexpressing indicated FLAG-HA-FAM134 proteins. 200 nM Bafilomycin A1 was added 2 h prior cell lysis.

H, I Representative Western blot of total cell lysates derived from wild-type (WT) MEFs and indicated *Fam134* knockout MEFs overexpressing the indicated Fam134 protein.

Source data are available online for this figure.

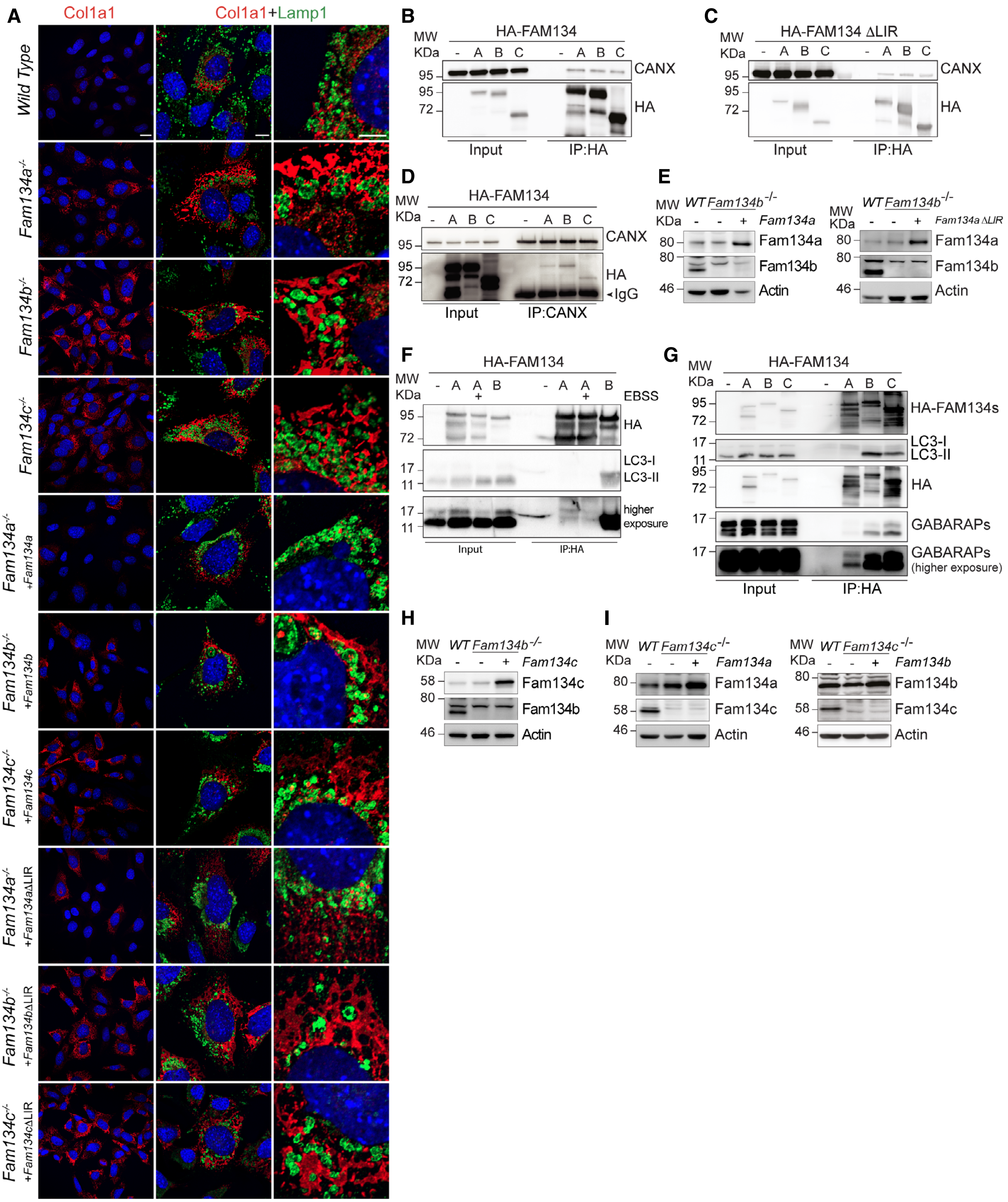

Figure EV5.

 © 2021 The Authors
