# Supplementary material for: Role of FAM134 paralogues in endoplasmic reticulum remodeling, ER‐phagy, and Collagen quality control
Source: EMBO Rep. 2021 Aug 2;22(9):e52289. doi: 10.15252/embr.202052289 (PMC8447607; doi:10.15252/embr.202052289)
Supplement: Supplementary file 13 — Movie EV2 [file EMBR-22-e52289-s013.zip › MovieEV2/MovieEV2_legend.docx]

**Movie EV2**

Simulation of FAM134B RHD changes in shape and conformation in a bilayer environment
