# Supplementary material for: Role of FAM134 paralogues in endoplasmic reticulum remodeling, ER‐phagy, and Collagen quality control
Source: EMBO Rep. 2021 Aug 2;22(9):e52289. doi: 10.15252/embr.202052289 (PMC8447607; doi:10.15252/embr.202052289)
Supplement: Supplementary file 15 — Movie EV4 [file EMBR-22-e52289-s016.zip › MovieEV4/MovieEV4_legend.docx]

**Movie EV4**

In silico membrane curvature-induction assay for FAM134A RHD embedded in discontinuous bicelle patches (DMPC+DHPC lipids)
