# Supplementary material for: Role of FAM134 paralogues in endoplasmic reticulum remodeling, ER‐phagy, and Collagen quality control
Source: EMBO Rep. 2021 Aug 2;22(9):e52289. doi: 10.15252/embr.202052289 (PMC8447607; doi:10.15252/embr.202052289)
Supplement: Supplementary file 19 — Movie EV8 [file EMBR-22-e52289-s004.zip › MovieEV8/MovieEV8_legend.docx]

**Movie EV8**

Time lapse of representative total imagine views of U2OS cells, expressing the ER-phagy reporter ssRFP-GFP-KDEL, treated with Torin1
