# Supplementary figures and images for: Role of FAM134 paralogues in endoplasmic reticulum remodeling, ER‐phagy, and Collagen quality control
Source: EMBO Rep. 2021 Aug 2;22(9):e52289. doi: 10.15252/embr.202052289 (PMC8447607; doi:10.15252/embr.202052289)

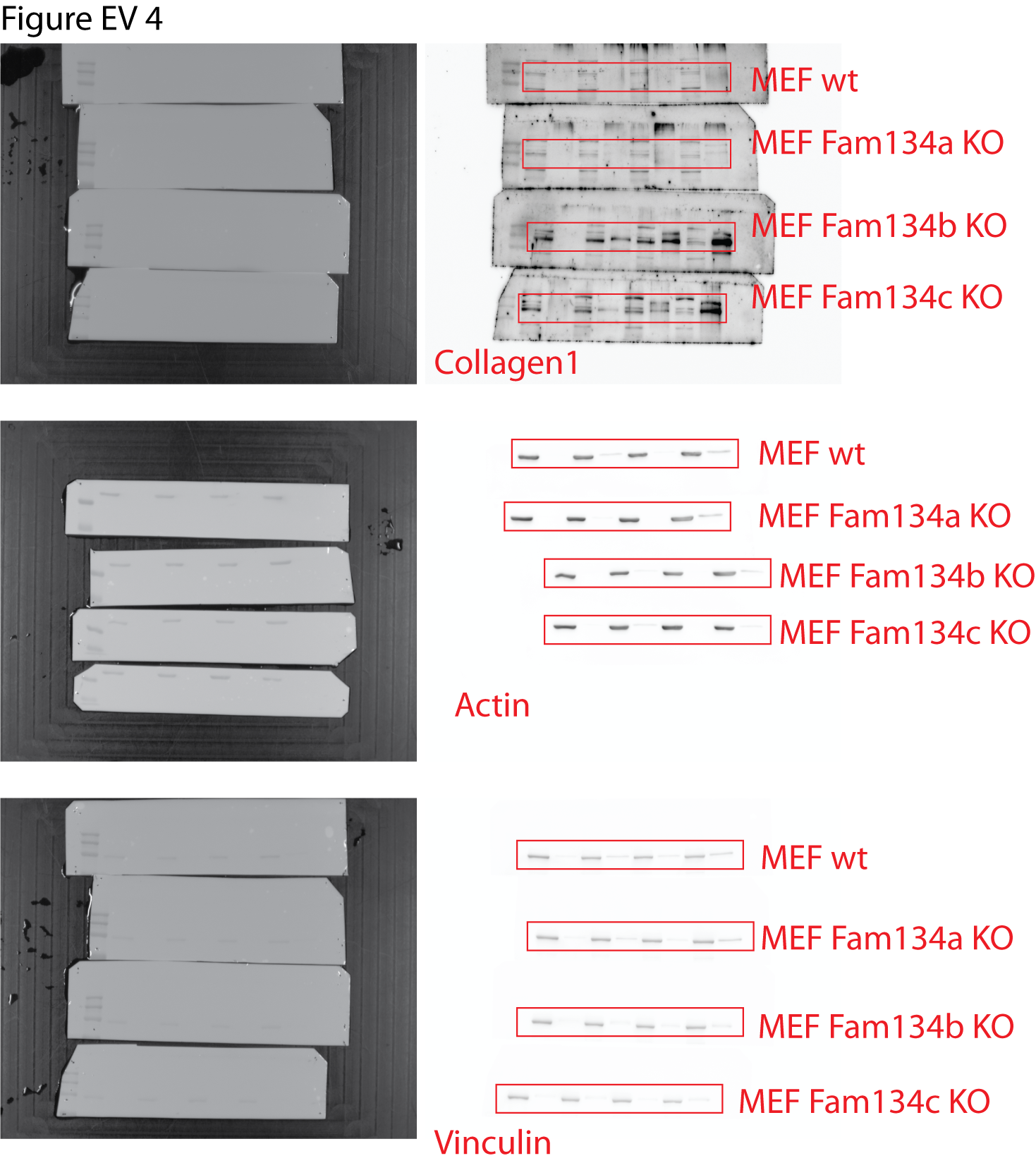

Supplement: Supplementary file 20 — Source Data for Expanded View [file EMBR-22-e52289-s006.zip › EV_Figure_Source_Data/Figure_EV4.tif]

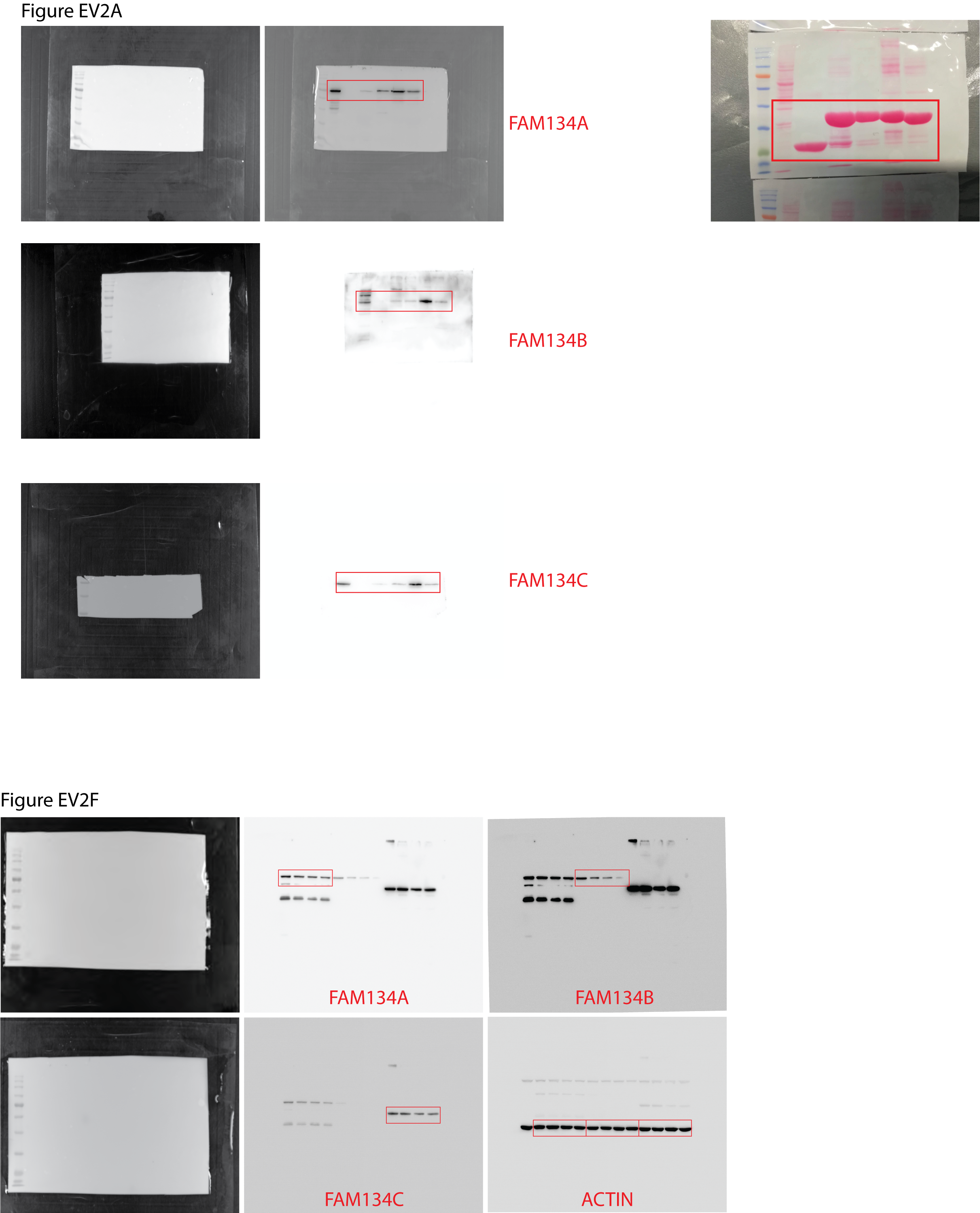

Supplement: Supplementary file 20 — Source Data for Expanded View [file EMBR-22-e52289-s006.zip › EV_Figure_Source_Data/Figure_EV2.tif]

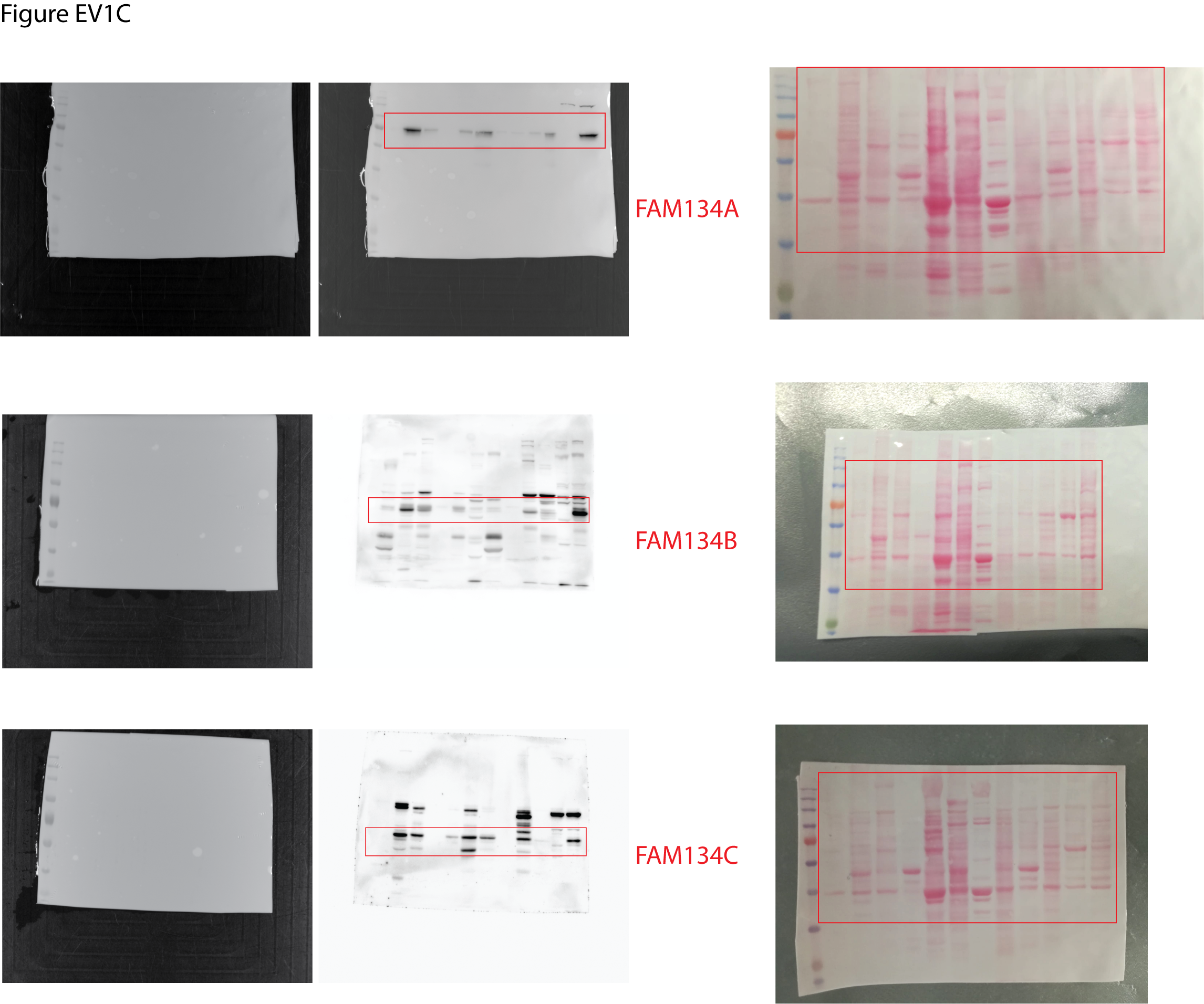

Supplement: Supplementary file 20 — Source Data for Expanded View [file EMBR-22-e52289-s006.zip › EV_Figure_Source_Data/Figure_EV1.tif]

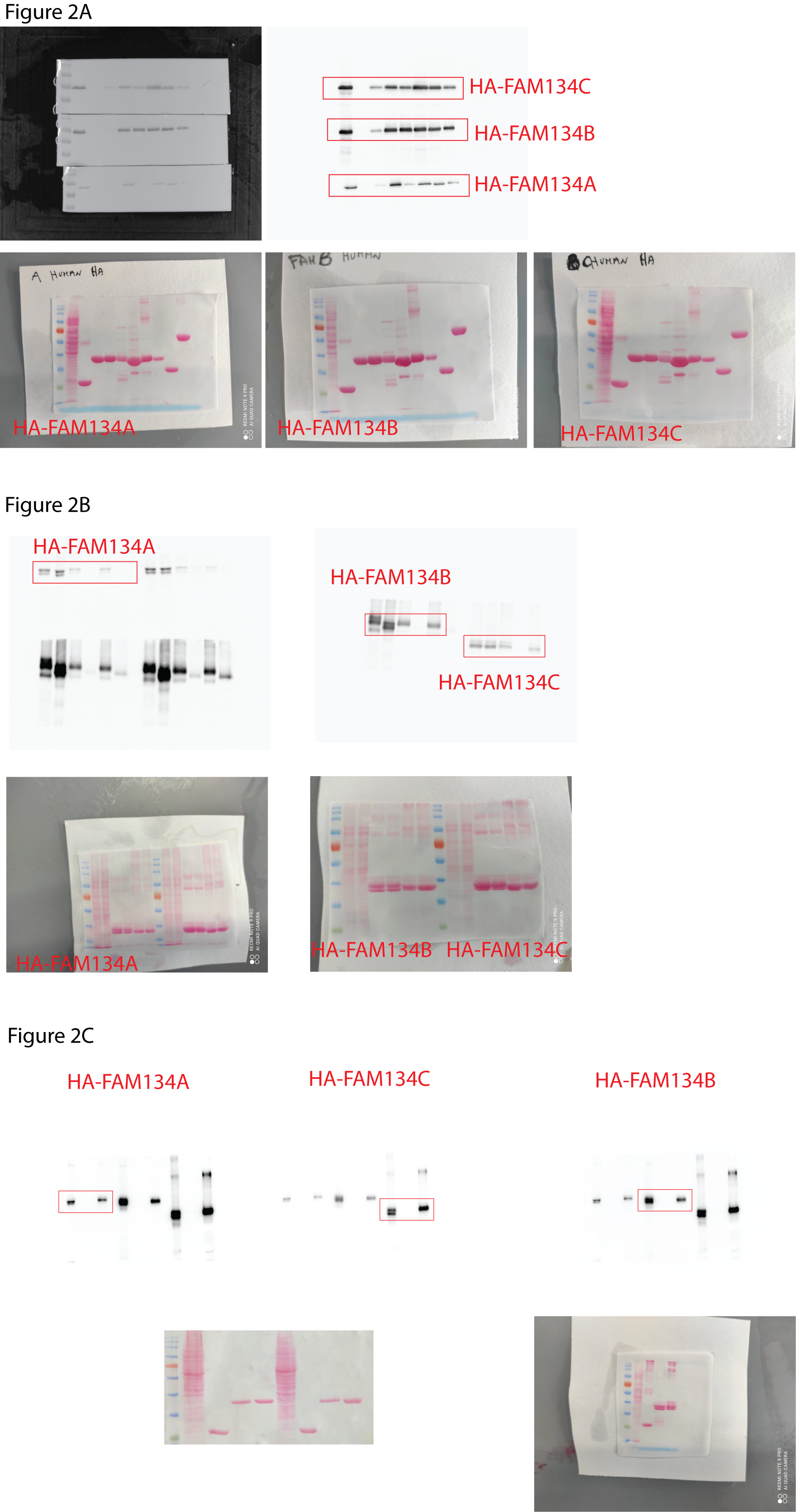

Supplement: Supplementary file 21 — Source Data for Figure 2 [file EMBR-22-e52289-s009.tif]

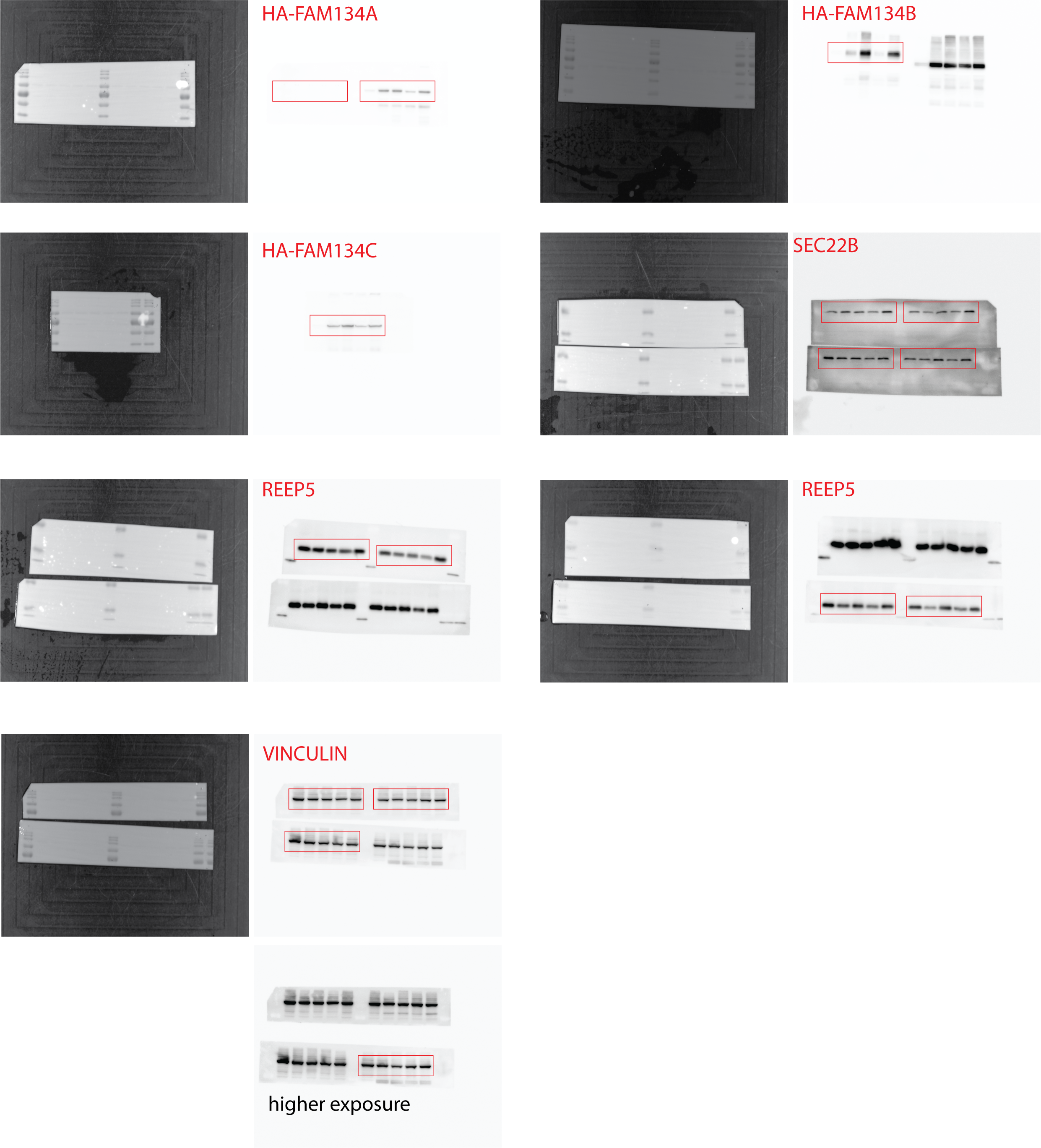

Supplement: Supplementary file 22 — Source Data for Figure 3 [file EMBR-22-e52289-s020.tif]

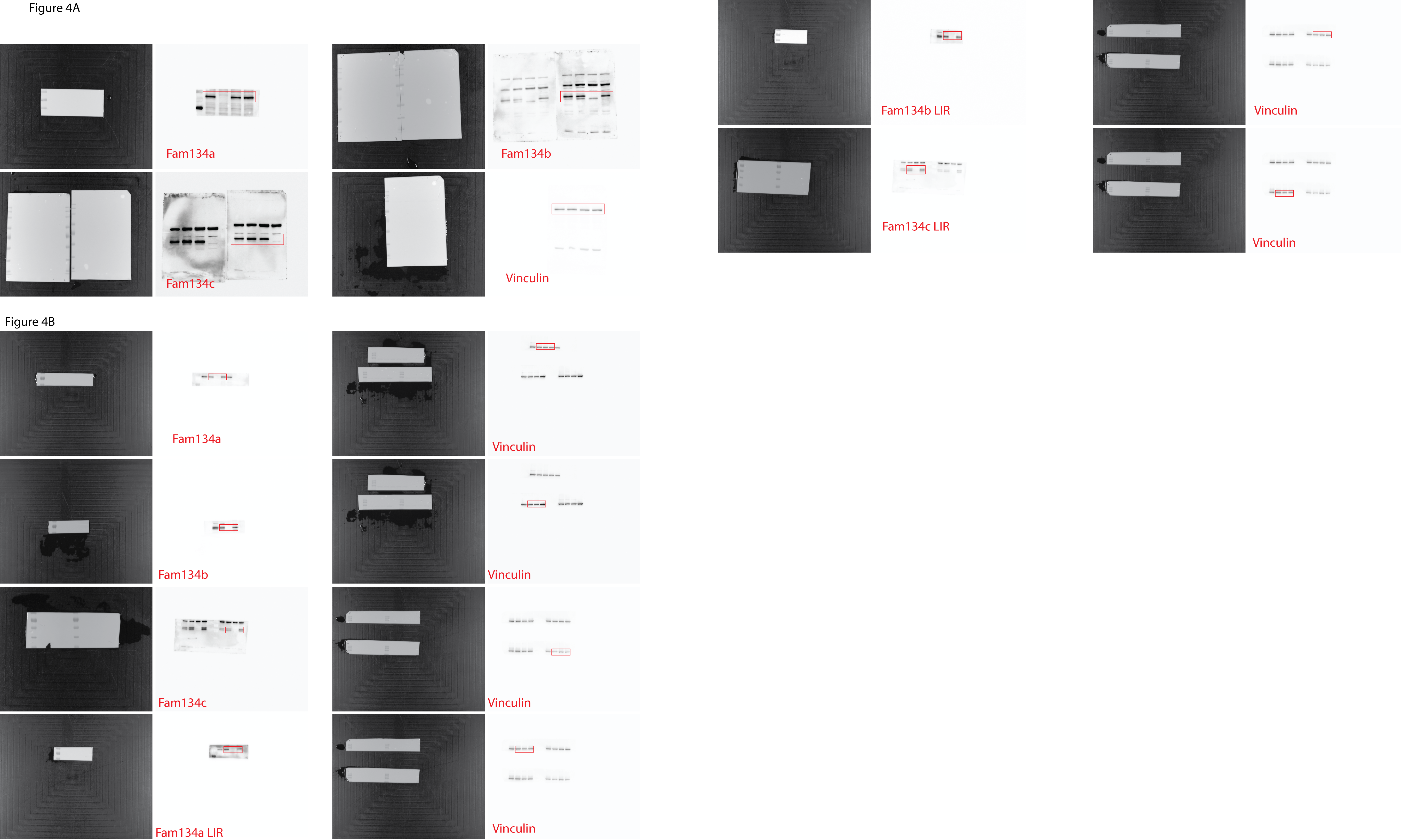

Supplement: Supplementary file 23 — Source Data for Figure 4 [file EMBR-22-e52289-s021.tif]

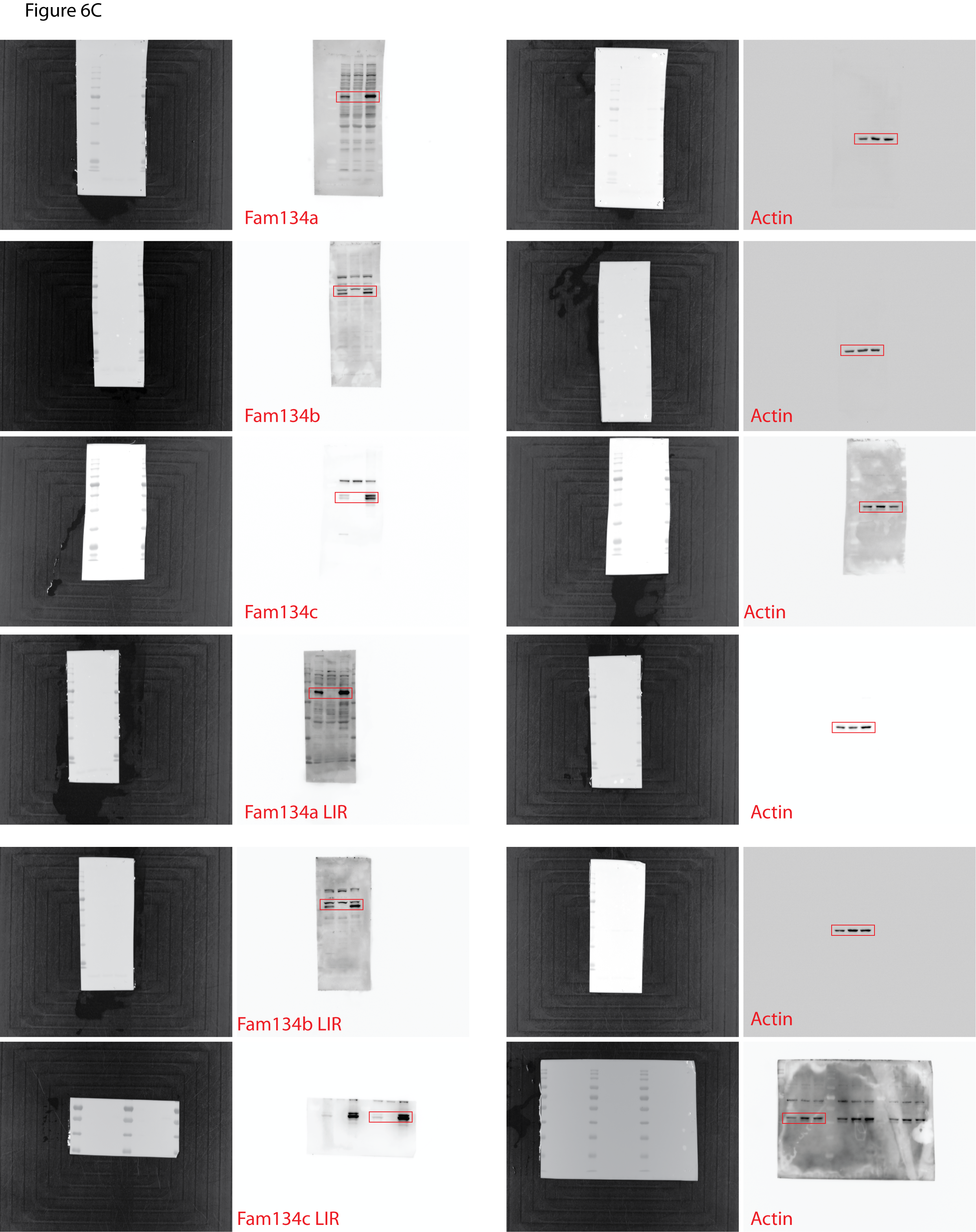

Supplement: Supplementary file 24 — Source Data for Figure 6 [file EMBR-22-e52289-s010.tif]

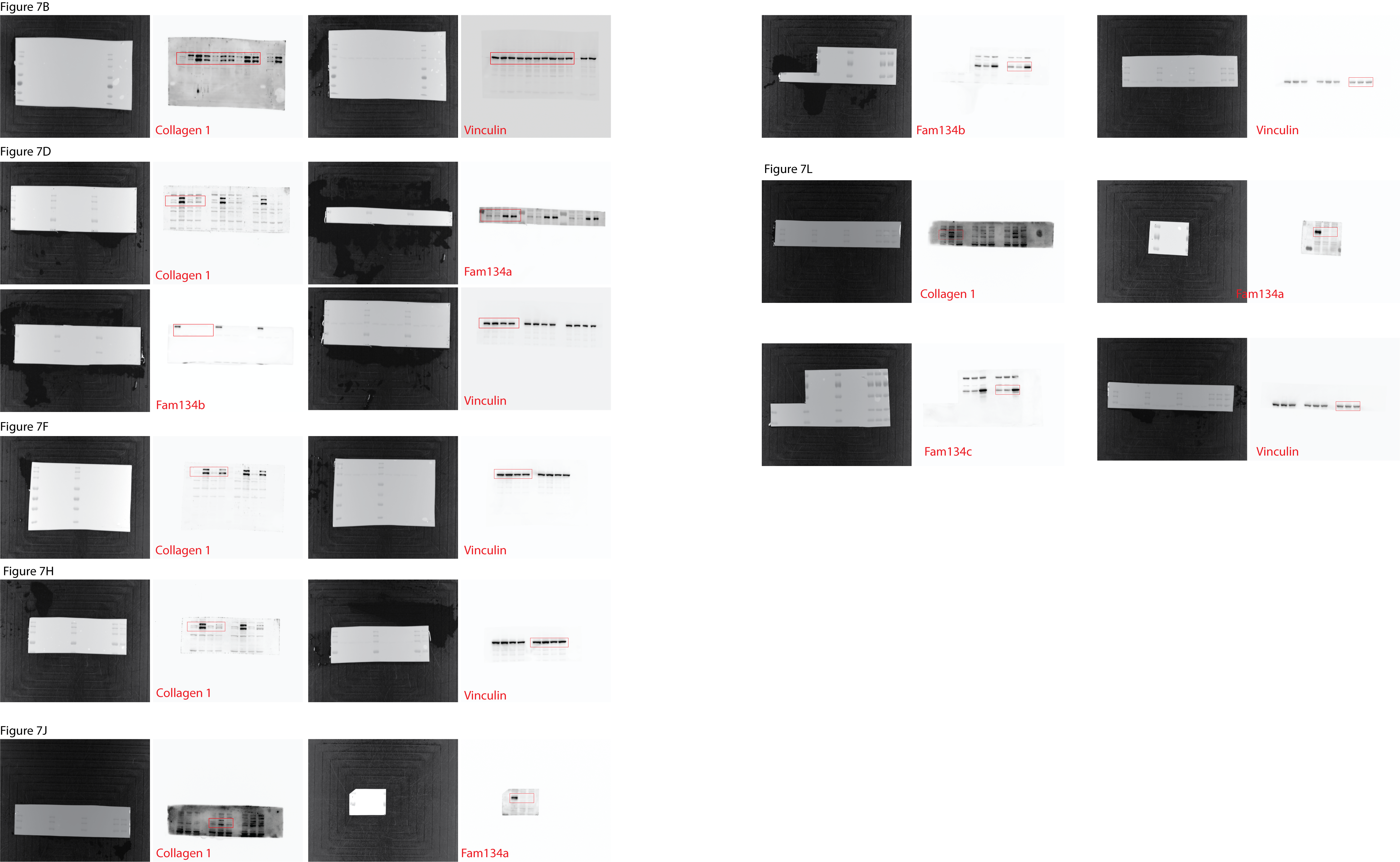

Supplement: Supplementary file 25 — Source Data for Figure 7 [file EMBR-22-e52289-s001.tif]
